# Latent profile analysis of nurse burnout categories and influencing factor differences among nurses in Southwest China

**DOI:** 10.3389/fpubh.2026.1764970

**Published:** 2026-03-23

**Authors:** Xia Li, Fengling Yang, Xingyu Chen, Huiling Zhao, Rong Yu

**Affiliations:** 1 Otolaryngology-Head and Neck Surgery Department of West China Hospital, Sichuan University, Chengdu, China; 2 Otolaryngology-Head and Neck Surgery Mianyang Central Hospital University of Electronic Science and Technology of China School of Medicine, Mianyang, China

**Keywords:** burnout, cross-sectional study, latent profile analysis, nurse work environment, nurses

## Abstract

**Objective:**

This study employs latent profile analysis (LPA) to identify potential categories of nurse burnout and to analyze differences in characteristics and influencing factors across burnout categories.

**Methods:**

From June to August 2025, a mixed sampling approach combining convenience and snowball sampling was used to recruit nurses from hospitals of varying levels in Southwest China. Three tools were used for data collection: A self-designed routine information questionnaire, Maslach Burnout Inventory-General Survey (MBI-GS) and Practice Environment Scale of the Nursing Work Index (PES-NWI), LPA identifies potential categories of nurses' professional burnout and uses multivariate logistic regression analysis to explore the factors associated with these categories.

**Results:**

This study comprised a total of 809 participants. LPA identified four distinct latent classes of nursing burnout: Class 1, low-burnout-high-efficacy (11.5%); Class 2, mild-burnout-unfulfilled (33.9%); Class 3, moderate-burnout-exhausted (44.6%); and Class 4, severe-burnout-dysfunctional (10.0%). Multivariate logistic regression analysis showed that age, years of work experience, hospital level, nurses' participation in hospital management, nursing quality standards, staffing and resource adequacy, and medical care cooperation are significant predictors of burnout among nurses (*P* < 0.05).

**Conclusion:**

Nurse burnout in southwest China is mainly moderate to severe and exhibits distinctive characteristics. It is recommended to implement personalized interventions tailored to the specific characteristics of nurses‘ professional burnout to alleviate the situation. Particular attention should be given to nurses with fewer than five years of experience by providing enhanced job support and psychological assistance to help them navigate critical periods of professional burnout. These measures aim to safeguard nurses' physical and mental health, improving the overall quality of nursing, and promoting the healthy development of global medical care.

## 1 Introduction

The revised eleventh edition of the World Health Organization's International Classification of Diseases (ICD-11, 2021) identifies occupational burnout as a serious health problem (1, 2). A meta-analysis examining nurse burnout across 30 countries revealed a global prevalence rate of 30%, with an increasing trend (3). In a survey of 2,000 nurses (4), about half of the respondents reported moderate to severe professional burnout. Despite differences in study results, nurse burnout remains a problem that cannot be ignored.

Maslach and Leiter defined occupational burnout as a negative psychological syndrome that stems from the individual's inability to cope with long-term occupational pressure effectively. This state is mainly manifested in changes in three areas: emotional exhaustion, depersonalization, and a weakening of the sense of accomplishment (5, 6). Occupational burnout is manifested as a series of symptoms, including irritability and anxiety in the workplace, as well as reduced patience and empathy for patients. In addition, research shows that this condition can cause a variety of physical health problems, including but not limited to sleep disorders, gastrointestinal dysfunction, and increased risk of cardiovascular disease (7, 8). When nurses are overly tired from work, the consequences are not only harmful to individuals but also to the whole medical system. For example, it leads to increased employee turnover and decreased job satisfaction, thereby endangering the long-term stability of the nursing team (9, 10). Moreover, it reduces the quality of hospital care services and endangers the effectiveness and safety of patients' treatment (10–12). A large number of studies have been conducted at home and abroad to investigate the current state of burnout among nurses and its related factors. These studies cover surveys conducted in hospitals at different levels and across various clinical departments (13–17). Numerous studies have demonstrated that, while accounting for sociodemographic variables including age, gender, and educational background (18, 19), the nursing environment and organizational support are significant determinants of burnout levels (20). However, there is still a lack of research on the unique potential characteristics of occupational burnout in the nursing environment and its related influencing factors. This study aims to use latent profile analysis (LPA) to systematically and quantitatively evaluate the possible categories of professional burnout of nurses in southwest China. Exploring the characteristics of different groups and their influencing factors provides a basis for formulating targeted intervention strategies to reduce nurses' professional burnout.

## 2 Methods

### 2.1 Study participants

This study employed a mixed convenience and snowball sampling approach. Participants were recruited from June to August 2025 among nurses at hospitals in Southwest China. The inclusion criteria were as follows: (1) possession of a valid qualification certificate, and (2) employment duration ≥ 1 year, with ≥ 6 months of nursing practice (including nursing management and clinical nursing roles) within the current year. The exclusion criterion was a history of psychiatric disorders or impaired consciousness. Based on a rough sample size calculation method, the sample should be at least 5–10 times the number of independent variables. Preliminary estimates for this study suggest a required sample size of 460 participants, factoring in an estimated 15% attrition rate, the target sample size should be at least 550 participants. Ethical approval for the research has been granted by West China Hospital of Sichuan University (approval no. 2025.2478). Participation was voluntary and anonymous.

### 2.2 Study tools

#### 2.2.1 Demographics

Demographic characteristics encompassed gender, age, educational level, status, years of service, hospital level, work unit, professional title, administrative position, yearly income, marital status, and living arrangements.

#### 2.2.2 Maslach Burnout Inventory-General Survey (MBI-GS)

Nurse burnout symptoms were measured by the Maslach Burnout Inventory-General Survey (MBI-GS) (5). The scale was developed by Maslak and cross-culturally verified by Li (21). MBI-GS contains three component tables - emotional exhaustion (questions 1–5), depersonalization (questions 6–9), and personal sense of achievement (questions 10–15), with a total of 15 items. Each item uses a 7-point Likert scale from 0 (never) to 6 (daily); higher scores on the “emotional exhaustion” and “depersonalization” dimensions indicate more severe professional burnout. “The higher the score of the 'low sense of achievement', the lighter the professional burnout. In our sample, the Cronbach's α coefficients for the subscales ranged from 0.831 to 0.950.

#### 2.2.3 Practice Environment Scale-Nursing Work in the Community (PES-NWI)

Practice Environment Scale of the Nursing Work Index (PES-NWI) is used to assess nurses' perceptions of their work environment. The scale was developed by Lake (22) and subsequently adapted and revised into a Chinese version by Wang Li (23). The scale contains 31 items, covering five dimensions: nurses' participation in hospital affairs (9 items), the basis of nursing quality (10 items), nurse management ability, leadership support for nurses (five items), staffing and resource adequacy (four items), and medical-care collaboration (three items). There are four answer options for each item, from “strongly oppose” to “strongly agree”, corresponding to 1–4 points. The higher the score, the better the nursing working environment. The Cronbach's alpha coefficient for each component table ranges from 0.67 to 0.79. In this analysis, the range of Cronbach's α coefficients in each dimension is 0.863–0.947.

### 2.3 Data collection

This study uses the Questionnaire Star Platform (www.wjx.cn), an online survey tool widely used in China. This research developed an electronic questionnaire, which was distributed via a WeChat link to the WeChat group of nursing professionals in southwest China. The questionnaire elaborates on the study's purpose and methods, and explains the potential risks to participants to ensure informed consent. All questions are required, and each IP address can only be submitted once.

### 2.4 Statistical analysis

Latent Profile Analysis (LPA) was conducted using Mplus 8.3 software, with the three subscale scores of the MBI-GS serving as the indicator variables. Model fit indicators include the Akaike information criterion (AIC), Bayesian information criterion (BIC), sample-size adjusted BIC (aBIC), entropy, the Lo–Mendell–Rubin adjusted likelihood ratio test (LMR), and the bootstrap likelihood ratio test (BLRT). A lower AIC, BIC, and aBIC values indicate a better fit. Entropy measures the model's accuracy; values closer to 1 indicate more precise classification.

LMR and BLRT are used to compare model-fitting differences; when the *p*-value is < 0.05, the model with k contours is better than the model with k-1 contours. Additionally, the smallest category should account for at least 10% of the total sample size.

SPSS 26.0 was used for statistical analysis. Categorical data are presented as frequencies and percentages. Based on the optimal model, chi-square tests and one-way ANOVA were used to compare differences in general information and nursing work environments across the latent categories. Multivariate logistic regression was used for multifactor analysis. P < 0.05 is considered statistically significant.

## 3 Results

### 3.1 Demographic characteristics of the study sample

A total of 820 questionnaires were recovered in this survey, including 809 valid questionnaires, with an efficiency of 98.7%. The remaining 11 questionnaires were eliminated for the following reasons: five submissions were incomplete or invalid, three were logically contradictory, and the completion time of another three was far below the average. The results showed that the participants were mainly women (95.9%), and a small proportion (4.1%) were men. Regarding job rank, junior educators accounted for the most significant proportion of participants (42.5%). In terms of education level, nurses with a bachelor's degree accounted for the main proportion of participants (77.9%). Regarding marital status and living arrangements, approximately 60% of participants were married with children, and 46.8% lived with their parents. Most nurses worked in surgical units (58.0%), followed by internal medicine (16.8%). Most participants (54.4%) were in the 20–58 years age group, with a mean age of 33.09 years (*SD* = 7.097). Additional sociodemographic characteristics of the study group are summarized in Table 1.

**Table 1:** Comparisons of Participants' sociodemographic characteristics and factors influencing categories of nurse burnout.

**Variable**	**Group**	***n* (%)**	**High sense of accomplishment - mild burnout (*n*)**	**Low sense of accomplishment - mild burnout (*n*)**	**High depersonalization - moderate burnout (*n*)**	**Low emotional - severe burnout (*n*)**	**χ^2^/F**	***P* 值**
**Gender**	Male	33 (4.1)	2	10	16	5	1.978	0.572
	Female	776 (95.9)	91	264	345	76		
**Age**	≤ 25	108 (13.3)	17	37	40	14	23.896	0.004^*^
	26–35	456 (56.4)	61	138	211	46	–	–
	36–45	194 (24.0)	12	71	94	17	–	–
	≥46	51 (6.3)	3	28	16	4	–	–
**Educational level**	Associate degree	151 (18.7)	24	46	70	11	8.740	0.189
	Bachelor's degree	630 (77.9)	68	216	277	69	–	–
	Master's degree or higher	28 (3.5)	1	12	14	1	–	–
**Employment status**	Short-term contract	246 (30.4)	30	84	113	19	7.187	0.304
	Long-term contract	411 (50.8)	53	134	177	47	–	–
	Permanent staff	152 (18.8)	10	56	71	15	–	–
**Years of services**	≤ 2 year	81 (10.0)	13	20	38	10	21.932	0.009^*^
	3–5 year	127 (15.7)	20	53	48	6	–	–
	5–10 year	247 (30.5)	32	70	116	29	–	–
	≥10 year	354 (43.8)	28	131	159	36	–	–
**Hospital level**	Community/secondary hospitals	71 (8.8)	10	11	42	8	12.019	0.007^*^
	Tertiary hospitals	738 (91.2)	83	263	319	73	–	–
**Work unit**	Internal medicine	136 (16.8)	18	44	55	19	9.819	0.365
	Surgical units	469 (58.0)	55	160	216	38	–	–
	Emergency/critical care/operating room	111 (13.7)	8	35	55	13	–	–
	others	93 (11.5)	12	35	35	11	–	–
**Professional title**	Nurse	125 (15.5)	21	38	48	18	28.438	< 0.001^*^
	Junior nurse	344 (42.5)	51	117	145	31	–	–
	Intermediate nurse	270 (33.4)	16	86	142	26	–	–
	Senior nurse	70 (8.7)	5	33	26	6	–	–
**Administ‘rative position**	Yes	104 (12.9)	6	40	52	6	7.067	0.070
	No	705 (87.1)	87	234	309	75	–	–
**Yearly income**	< 5,0000 RMB	68 (8.4)	9	17	33	9	27.061	0.001^*^
	50,000–100,000 RMB	357 (44.1)	50	100	171	36		
	100,000–200,000 RMB	332 (41.0)	32	128	137	35		
	Above 200,000 RMB	52 (6.4)	2	24	20	1		
**Marital status**	Unmarried	211 (26.1)	35	67	85	24	8.549	0.036^*^
	Married	71 (73.9)	58	207	276	57		
**Living arrangements**	Living alone	109 (13.5)	19	29	50	11	12.536	0.185
	Shared living	67 (8.3)	12	24	24	7		
	Independent household (not living with parents)	254 (31.4)	26	88	110	30		
	Living with parents	379 (46.8)	36	133	177	33		
Nurse participation in hospital affairs (x ± s)			1.691 ± 0.257	1.742 ± 0.267	2.210 ± 0.206	2.384 ± 0.485	70.400	< 0.001^*^
Nursing foundations for quality of care (x ± s)			1.600 ± 0.224	1.561 ± 0.218	1.986 ± 0,156	2.019 ± 0.307	59.640	< 0.001^*^
Nurse manager ability, leadership, and support of nurses (x ± s)			1.626 ± 0.264	1.623 ± 0.229	2.068 ± 0.197	2.212 ± 0.511	61.912	< 0.001^*^
Staffing and resource adequacy (x ± s)			1.677 ± 0.336	1.777 ± 0.311	2.266 ± 0.248	2.574 ± 0.580	77.159	< 0.001^*^
Collegial nurse – physician relations (x ± s)			1.627 ± 0.275	1.572 ± 0.234	2.011 ± 0.197	2.169 ± 0.434	59.518	< 0.001^*^

^*^Statistically significant, *p*-value < 0.05.

### 3.2 Latent profile determination

LPA models with one to six classes were constructed using the MBI-GS three-dimensional scores as the observation indicators. The AIC, BIC, and aBIC values decreased progressively from the 1-class model to the 6-class model, indicating improved model fit. All the models had entropy values >0.8, indicating good classification quality. The four-class model had LMR and BLRT *P*-values < 0.01, and the entropy value (0.845) was lower than that of the five-class model (0.861). Although the 3-class and 5-class models passed the statistical tests (*P* < 0.05), they included a small probability category (< 10% of the sample) (24). The detailed model fit statistics are presented in Table 2.

**Table 2:** Fit indices of latent profile analyses (Classes 1–6, *N* = 809).

**Model**	**AIC**	**BIC**	**aBIC**	**Entropy**	**P**		**Classprobabilities**
					**LMRT**	**BLRT**	
1	16279.401	16307.576	16288.523	–	–	–	1
2	15648.228	15695.186	15663.431	0.912	< 0.001	< 0.001	0.84/0.16
3	15324.948	15390.689	15346.231	0.840	0.0005	< 0.001	0.54/0.09/0.38
4	15110.889	15195.414	15138.253	0.845	0.0001	< 0.001	0.11/0.45/0.37/0.10
5	14919.093	15022.401	14952.538	0.861	0.0000	< 0.001	0.32/0.41/0.11/0.04/0.12
6	14861.77	14983.861	14901.296	0.829	0.2420	< 0.001	0.26/0.30/0.57/0.12/0.23/0.04

AIC, Akaike information criterion; BIC, Bayesian information criterion; aBIC, adjusted Bayesian information criterion; LMRT, Lo–Mendell–Rubin likelihood ratio test; BLRT, bootstrap likelihood ratio test.

**Figure 1:**
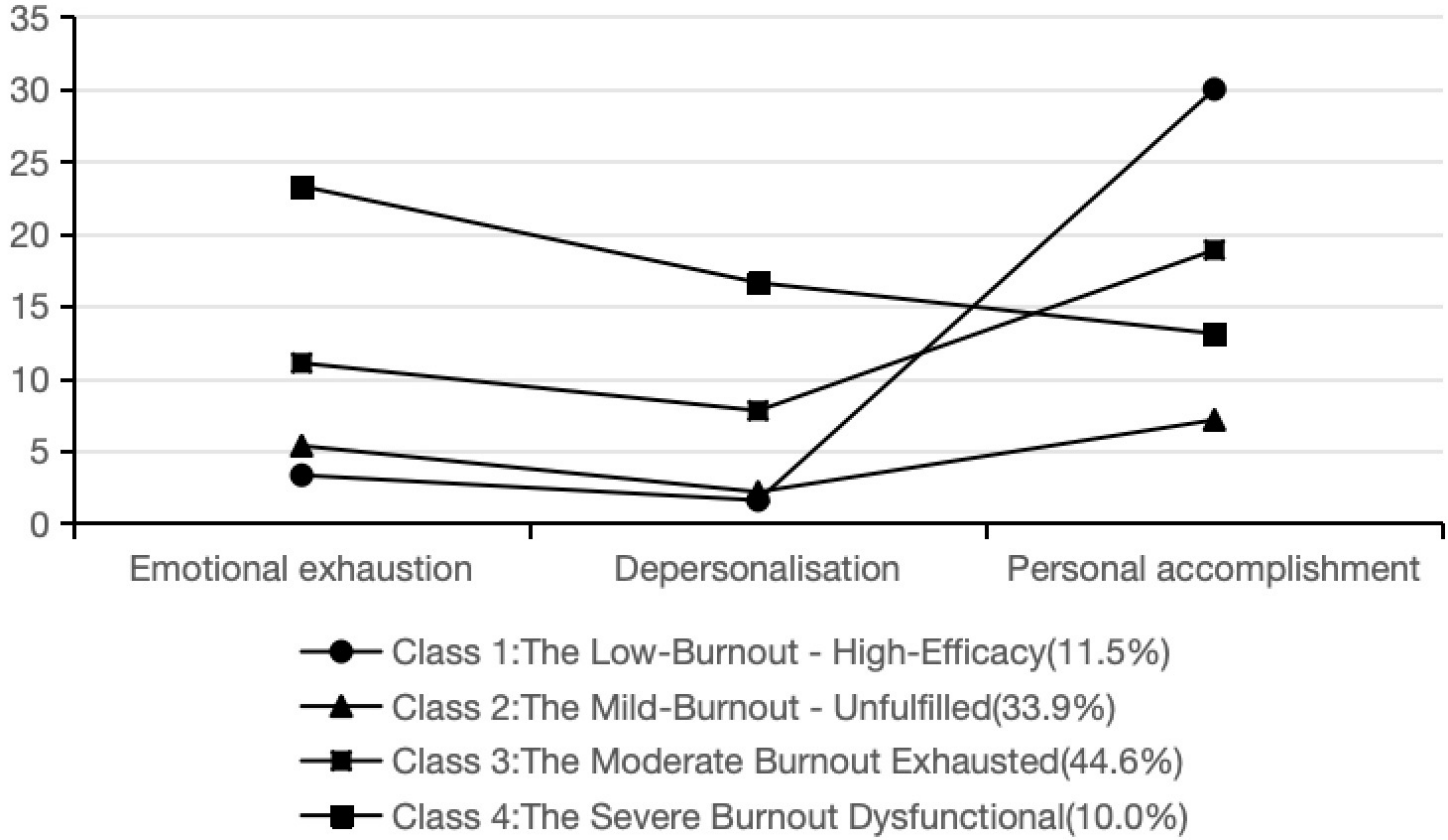
Potential profiles of nursing burnout

This study analyzes the characteristics of four potential categories of nurse burnout by plotting a line chart of scores across various dimensions of nursing professional burnout (Figure 1). based on the external features of each dimension of the scale, these categories are as follows: Class 1 includes 93 cases (11.5%), where mild burnout is present, particularly where nurses exhibit mild occupational burnout, particularly when they derive a sense of accomplishment from their work and feel confident in completing routine nursing tasks; consequently, this profile is designated “Low Burnout–High-Efficacy”. Class 2 includes 274 cases (33.9%) in which nurses exhibit mild occupational burnout. Despite their mild severity, these individuals demonstrated poor work accomplishment, primarily manifested as low confidence in completing their tasks and diminished ability to derive value from their work. Consequently, this profile was designated “Mild Burnout-Unfulfilled”. Class 3 included 361 cases (44.6%). When nurses in this group experienced moderate burnout, they occasionally viewed their work as a compulsory task, disregarding its meaning, and maintaining emotional detachment from others; hence, it was named Moderate Burnout-Exhausted (44.6%), Class 4 includes 81 patients (10.0%), who experienced severe occupational burnout, particularly characterized by frequent feelings of fatigue during work and a perception that work demands are overwhelming; hence, it is named “Severe Burnout- Dysfunctional”.

### 3.3 Factors associated with potential profiles of nurse burnout

Univariate analysis revealed that the four burnout profile categories among nurses differed significantly by age (*P* < 0.001), professional title (*P* < 0.001), hospital level (*P* = 0.011), marital status (*P* =0.014), years of service (*P* = 0.009), nursing work environment was characterized by five distinct dimensions: nurse participation in hospital affairs (*P* < 0.001); nursing foundations for quality of care (*P* < 0.001); nurse manager ability, leadership and support of nurses (*P* < 0.001); staffing and resource adequacy (*P* < 0.001); and collegial nurse–physician relationships (*P* < 0.001), as shown in Table 1.

### 3.4 Multivariate logistic regression analysis of potential classes of nurse burnout

A multivariate logistic regression model was constructed, incorporating statistically significant variables from the univariate analysis as the dependent variable.

The multivariate analysis results indicate that age, years of service, hospital level, nurse participation in hospital affairs, nursing foundations for quality of care, staffing and resource adequacy, and collegial nurse–physician relationships are factors influencing nurses' professional potential categories. Compared to nurses in the “Low-Burnout - High-Efficacy” (Class 1), those working in community or secondary hospitals were more likely to be in Class 1 burnout than those in tertiary hospitals (*P* = 0.033). Nurses with annual incomes of 50,000–100,000 RMB were also more likely to be in this class than those earning over 200,000 RMB (*P* = 0.050). Additionally, nurses with 3–5 years of work experience (*P* = 0.035) were more likely to experience Class 1 burnout compared to those in “The Severe Burnout Dysfunctional” (Class 4). In the comparison between Class 2 and “The Moderate Burnout Exhausted” (Class 3), individuals aged 26–35 (*P* = 0.029) and 36–45 (*P* = 0.046) were more likely to belong to Class 3 burnout than those aged 46 or older. Compared with Class 4, nurses with 3–5 years of work experience (*P* = 0.001) were more likely to belong to Class 2 burnout; nurses with ≤ 2 years (*P* = 0.019) and 3–5 years (*P* = 0.003) of work experience were more likely to belong to Class 3 burnout. The nursing professional environment influences different latent categories. In terms of the outcome of nurse participation in hospital affairs, significant positive correlations were observed in comparisons between “The Low-Burnout - High-Efficacy”(Class 1) and Class 2 burnout (*P* = 0.024), Class 1 and Class 3 burnout *(P* = 0.005), and Class 1 and Class 4 burnout (*P* = 0.002). The study found that the higher the degree of participation of nurses in hospital affairs, the greater the possibility of belonging to Class 1 professional burnout (compared to Class 2, 3, and 4). The comparison between Class 3 and Class 4 (*P* = 0.031) shows that the higher the basic score of nursing quality, the more likely the nurse is to belong to the Class 3 career burnout category. In terms of staffing and resource adequacy, Class 1 and Class 2 (*P* = 0.019), Class 1 and Class 3 (*P*=0.001), Class 1 and Class 4 (*P* < 0.001), Class 2 and Class 4 (*P* < 0.001), Class 3 and Class 4 (*P* < 0.001), There is a significant positive correlation between them. When the staffing and resources of nurses are more sufficient, the more likely it is that nurses are in Class 1, Class 2, or Class 3 burnout categories. Regarding the cooperative relationship between nurses and doctors, the comparison between Class 1 vs. Class 2 (*P* = 0.046) and Class 2 vs. Class 4 (*P* = 0.057) shows that a more harmonious medical relationship with nurses is more prone to Class 1 or Class 2 professional burnout, as shown in Table 3.

**Table 3:** Multivariate regression results of the profile types of occupational burnout.

**Variables**	**Classification**	**Class 1 vs. Class 2**				**Class 1 vs. Class3**			
		**B**	***P***	**OR**	**95% CI**	**B**	***P***	**OR**	**95% CI**
Age (≥46 as a reference)	≤ 25	−0.494	0.62	0.610	(0.086, 4.306)	−0.106	0.917	0.899	(0.122, 6.608)
	26–35	−1.022	0.221	0.360	(0.07, 1.852)	0.013	0.988	1.013	(0.191, 5.383)
	36–45	−0.256	0.741	0.774	(0.169, 3.541)	0.584	0.463	1.794	(0.377, 8.547)
Years of service (≥10 year as a reference)	≤ 2 year	0.239	0.747	1.270	(0.297, 5.434)	1.131	0.118	3.098	(0.749, 12.814)
	3–5 year	0.781	0.137	2.183	(0.78, 6.106)	0.406	0.446	1.501	(0.528, 4.263)
	5–10 year	0.307	0.447	1.359	(0.616, 3.002)	0.356	0.379	1.428	(0.646, 3.156)
Hospital level (Tertiary hospitals as a reference)	Community/Secondary hospitals	−1.089	0.033^*^	0.337	(0.124, 0.914)	−0.606	0.176	0.546	(0.227, 1.312)
Professional title (senior nurse **as a reference**)	Nurse	−0.207	0.804	0.813	(0.158, 4.178)	−0.308	0.714	0.735	(0.141, 3.822)
	Junior nurse	−0.075	0.915	0.928	(0.237, 3.633)	−0.195	0.781	0.823	(0.208, 3.26)
	Intermediate nurse	0.428	0.516	1.534	(0.422, 5.577)	0.619	0.352	1.857	(0.505, 6.829)
Yearly income (Above 200,000 RMBas a reference)	< 50,000 RMB	−1.224	0.182	0.294	(0.049, 1.776)	−0.791	0.397	0.453	(0.073, 2.829)
	50,000–100,000 RMB	−1.552	0.05^*^	0.212	(0.045, 1.003)	−1.089	0.185	0.337	(0.067, 1.685)
	100,000–200,000 RMB	−0.945	0.227	0.389	(0.084, 1.801)	−0.813	0.318	0.444	(0.09, 2.188)
Marital Status (married as a reference)	Unmarried	−0.386	0.291	0.680	(0.332, 1.391)	−0.384	0.294	0.681	(0.333, 1.395)
Nurse participation in hospital affairs		1.490	0.024^*^	4.438	(1.218, 16.172)	1.827	0.005^*^	6.214	(1.738, 22.208)
Nursing foundations for quality of care		−1.076	0.134	0.341	(0.083, 1.392)	−0.368	0.605	0.692	(0.172, 2.784)
Nurse manager ability, leadership, and support of nurses		−0.555	0.395	0.574	(0.16, 2.064)	−0.236	0.711	0.790	(0.226, 2.756)
Staffing and resource adequacy		1.131	0.019^*^	3.100	(1.208, 7.955)	1.570	0.001^*^	4.808	(1.887, 12.249)
Collegial nurse-physician relations		−1.064	0.046^*^	0.345	(0.121, 0.982)	−0.512	0.32	0.599	(0.218, 1.644)
Age (≥46 as a reference)	≤ 25	1.207	0.382	3.343	(0.223, 50.123)	0.388	0.551	1.474	(0.412, 5.268)
	26–35	0.334	0.752	1.397	(0.175, 11.128)	1.035	0.029^*^	2.815	(1.114, 7.113)
	36–45	0.646	0.514	1.907	(0.275, 13.243)	0.841	0.046^*^	2.318	(1.015, 5.297)
Years of service (≥10 year as a reference)	≤ 2 year	−1.043	0.341	0.352	(0.041, 3.02)	0.892	0.123	2.439	(0.785, 7.578)
	3–5 year	−1.849	0.035^*^	0.157	(0.028, 0.878)	−0.375	0.348	0.687	(0.314, 1.504)
	5–10 year	0.068	0.896	1.070	(0.387, 2.96)	0.049	0.866	1.050	(0.595, 1.852)
Hospital level (Tertiary hospitals as a reference)	Community/secondary hospitals	−0.955	0.119	0.385	(0.116, 1.277)	0.483	0.225	1.621	(0.743, 3.539)
Professional title (senior nurse as a reference)	Nurse	1.135	0.297	3.112	(0.369, 26.287)	−0.101	0.86	0.904	(0.295, 2.77)
	Junior nurse	−0.025	0.977	0.975	(0.172, 5.534)	−0.120	0.777	0.887	(0.385, 2.041)
	Intermediate nurse	0.253	0.762	1.288	(0.25, 6.638)	0.191	0.612	1.210	(0.579, 2.531)
Yearly income (Above 200,000 RMBas a reference)	< 50,000 RMB	0.784	0.588	2.189	(0.128, 37.368)	0.433	0.412	1.542	(0.548, 4.338)
	50,000–100,000 RMB	0.402	0.761	1.495	(0.113, 19.826)	0.463	0.234	1.589	(0.741, 3.41)
	100,000–200,000 RMB	0.939	0.472	2.558	(0.197, 33.15)	0.133	0.724	1.142	(0.546, 2.387)
Marital Status (married as a reference)	Unmarried	−0.134	0.787	0.875	(0.331, 2.309)	0.003	0.993	1.003	(0.569, 1.766)
Nurse participation in hospital affairs		2.309	0.002^*^	10.065	(2.325, 43.561)	0.336	0.368	1.400	(0.673, 2.911)
Nursing foundations for quality of care		−1.409	0.081	0.244	(0.05, 1.189)	0.708	0.104	2.031	(0.864, 4.772)
Nurse manager ability, leadership, and support of nurses		−0.620	0.391	0.538	(0.13, 2.219)	0.319	0.403	1.376	(0.651, 2.909)
Staffing and resource adequacy		2.846	< 0.001^*^	17.227	(5.882, 50.453)	0.439	0.102	1.551	(0.917, 2.624)
Collegial nurse-physician relations		−0.260	0.657	0.771	(0.245, 2.425)	0.552	0.094	1.737	(0.91, 3.316)
Age (≥46 as a reference)	≤ 25	1.701	0.133	5.479	(0.594, 50.491)	1.313	0.223	3.718	(0.45, 30.694)
	26–35	1.356	0.083	3.881	(0.837, 17.99)	0.321	0.668	1.379	(0.318, 5.969)
	36–45	0.902	0.209	2.464	(0.603, 10.078)	0.061	0.93	1.063	(0.272, 4.154)
Years of service (≥10 year as a reference)	≤ 2 year	−1.282	0.2	0.277	(0.039, 1.969)	−2.174	0.019^*^	0.114	(0.019, 0.698)
	3–5 years	−2.629	0.001^*^	0.072	(0.015, 0.347)	−2.255	0.003^*^	0.105	(0.024, 0.467)
	5–10 years	−0.239	0.582	0.787	(0.336, 1.844)	−0.288	0.46	0.750	(0.349, 1.611)
Hospital level (tertiary hospitals as a reference)	Community/secondary hospitals	0.134	0.815	1.143	(0.373, 3.506)	−0.349	0.464	0.705	(0.277, 1.797)
Professional title (senior nurse as a reference)	Nurse	1.342	0.133	3.828	(0.663, 22.098)	1.443	0.08	4.234	(0.84, 21.338)
	Junior nurse	0.049	0.942	1.051	(0.276, 3.998)	0.170	0.791	1.185	(0.338, 4.156)
	Intermediate nurse	−0.175	0.781	0.839	(0.245, 2.875)	−0.366	0.54	0.694	(0.216, 2.232)
Yearly income (above 200,000 RMBas a reference)	< 50,000 RMB	2.008	0.1	7.448	(0.681, 81.466)	1.575	0.18	4.831	(0.482, 48.385)
	50,000–100,000 RMB	1.954	0.076	7.056	(0.817, 60.917)	1.491	0.166	4.440	(0.539, 36.591)
	100,000–200,000 RMB	1.884	0.083	6.582	(0.782, 55.383)	1.752	0.101	5.764	(0.713, 46.623)
Marital status (married as a reference)	Unmarried	0.252	0.566	1.287	(0.544, 3.045)	0.250	0.527	1.284	(0.592, 2.784)
Nurse participation in hospital affairs		0.819	0.113	2.268	(0.824, 6.238)	0.482	0.288	1.620	(0.666, 3.942)
Nursing foundations for quality of care		−0.332	0.561	0.717	(0.234, 2.2)	−1.041	0.031^*^	0.353	(0.137, 0.909)
Nurse manager ability, leadership, and support of nurses		−0.065	0.898	0.937	(0.347, 2.53)	−0.384	0.366	0.681	(0.296, 1.567)
Staffing and resource adequacy		1.715	< 0.001^*^	5.557	(2.68, 11.524)	1.276	< 0.001^*^	3.583	(1.875, 6.847)
Collegial nurse-physician relations		0.805	0.057	2.236	(0.975, 5.127)	0.252	0.484	1.287	(0.636, 2.606)

^*^Statistically significant, *p*-value < 0.05.

OR, odds ratio; CI, confidence Iinterval; the reference category is: Class 1: the low-burnout - high-efficacy; class 2: the mild-burnout - unfulfilled; Class 3: the moderate burnout exhausted; class 4: the severe burnout dysfunctional.

## 4 Discussion

At present, nurses worldwide continue to experience varying degrees of occupational burnout (3, 13, 25–27), which seriously affects the physical and mental health of nurses and the long-term healthy development of the medical system. Therefore, we need further to understand the potential categories of occupational burnout among nurses and implement individualized interventions and prevention strategies for each categoryThis study employed Latent Profile Analysis (LPA) to identify four unique burnout categories among nurses in Southwest China: Class 1: low burnout–high-efficacy (11.5%), Class 2: mild burnout–unfulfilled (33.9%), Class 3: moderate burnout-exhausted (44.6%), and Class 4: severe burnout-dysfunctional (10.0%). Approximately 10% of the nurses in this survey have moderate to severe occupational burnout, which is similar to the results of Lv and Ding et al. (28), but the overall result is lower than Ji et al. 17.1% (15). In the present report, emotional exhaustion and depersonalization are particularly prominent among nurses with moderate and severe burnout, consistent with previous studies and Rizzo et al. The results of systematic evaluation and meta-analysis are consistent (29, 30).

### 4.1 Factors influencing nursing burnout in nurses

Age: Nurses' age is associated with their level of nursing burnout. Compared with nurses aged 46 years or older, those aged 26–35 years and 36–45 years are more likely to fall into the moderate burnout-exhausted category. This finding is consistent with the results reported by Sabbah et al. (31). The prevalence of emotional burnout among the bachelor's degree-prepared nurses in this study may be attributed to their specific career stage. Since the composition of nurses in this study is mainly undergraduate graduates, they may have completed the new nurse stage and are now entering a period when they need to develop their professional skills and abilities further, prepare to start a family and raise children, and balance their relationships with family and work. At the same time, during this period, nurses are the main shift workers in the department, and their shifts are irregular, so they are more likely to experience emotional burnout, feel under greater pressure, and feel exhausted (32–34). Hospital level: Compared with nurses in tertiary hospitals, nurses in secondary and community hospitals are more likely to experience lower occupational burnout and a higher sense of professional effectiveness. Showed lower burnout, a higher sense of personal achievement, and stronger career satisfaction. This difference may be due to the more complex condition of patients usually admitted to tertiary hospitals. In addition, nurses in third-level hospitals are under greater pressure due to the multiple responsibilities of scientific research, teaching, and clinical work. The working environment and content of secondary and community hospitals are relatively simple (30, 35). Job tenure: The accumulation of work experience is closely related to nurses' age. Among new and junior nurses, professional burnout is low, manifesting as a “mild burnout-unsatisfied” state, mainly due to low professional effectiveness. The reason may be that they are enthusiastic about their careers at the beginning, but they feel unfamiliar with the work content. They cannot handle some emergencies on their own and are afraid of making mistakes. Therefore, they will not feel overly tired or indifferent toward patients at work, but they will lack confidence in their work and a sense of accomplishment. This differs from the research results of Lan et al., indicating that nurses experience different types of professional burnout at the beginning of their careers, which may also be influenced by factors such as region and group (36). At the same time, compared with senior nurses (≥10 years), middle-aged and younger nurses are more prone to moderate burnout-exhaustion, with emotional failure as the primary externalized manifestation. It may be that, as nurses gain more experience and age, their workload and responsibilities at work, as well as factors related to burnout, such as promotion and title changes, and pressure build, make it easy to feel unmotivated and tired. However, by this time, such nurses had mastered specific professional skills and gained work experience, so they had a sense of accomplishment in their work (37, 38). Conversely, some scholars have observed that as nurses gain more years of experience, their increased adaptability and resilience within the profession lead to lower levels of burnout than among younger nurses (36). Therefore, there are still different results and views on the types of professional burnout caused by working hours, which may be due to cultural and organizational backgrounds.

### 4.2 Impact of the nursing work environment on professional burnout

Regarding the impact of nurses' professional environment on burnout, many studies have shown that it affects burnout to varying degrees (39–41). The results show that across different dimensions of the nursing professional environment, nurse participation in hospital affairs, nursing foundations for quality of care, staffing and resource adequacy, and collegial nurse-physician relationships affect the potential categories of nurse professional burnout. Nurse manager ability, leadership, and support of nurses have no significant effect on the possible category of nurses' professional burnout, and scholars have reported similar results before (25, 42). In this study, no significant correlation was found between the head nurse's ability, leadership, and support and the potential category of professional burnout. The participation of nurses in hospital affairs is an organizational phenomenon that provides frontline nurses with opportunities to engage in internal hospital management and operations, as well as in decision-making related to policies and practices. Hence, when nurses are more involved in hospital affairs, their sense of pioneering spirit is stronger, leading to greater dedication to their work. This, in turn, reduces both emotional stress and physical fatigue while enhancing their sense of accomplishment and confidence. Consequently, they are more likely to be low-burnout-high-efficacy (43). “Sufficiency of staffing and resources” represents nurses' views on workload management. There is a strong connection between nurses' workload and all potential categories of professional burnout. Research results show that the higher the staffing and the availability of nurses' resources, the greater the likelihood of a milder form of occupational burnout (44). The increase in staffing and the adequacy of resources will reduce nurses' emotional fatigue, which, in turn, will further affect the disintegration of personality and personal achievement (45). Due to fatigue or work overload, nurses' professional burnout will increase, eventually leading to safety problems in nursing. (18). A high-quality nursing environment provides a foundation for hospital development and patient safety (39). The results of this study show that with a high-quality nursing quality assurance system and professional development support for nurses, the type of professional burnout among nurses is more likely to be the moderate burnout-exhausted than Class 4, but still moderate professional burnout. The hospital develops plans to support nurses' career development, regularly provides feedback on leadership skills evaluations, and redesigns workflows to reduce overload and task duplication. However, to maintain high-quality nursing care and services, hospitals may need to implement quality improvement plans continually. Although nurses have a positive attitude toward this, it is necessary to spend extra time on relevant activities and invest more energy in completing daily work, so nurses may still experience professional burnout even when the basic nursing quality score is high (46). A lack of support and respect in the practical environment within the medical team is considered an essential reason for burnout, and the results of this study also support this. Positive medical and nursing cooperation improves the environmental atmosphere perceived by nurses. Nurses are better able to complete their work proactively, strengthen their sense of identity with their work, and enhance their personal sense of achievement (47). Therefore, it is a protective factor for nurses' professional burnout.

### 4.3 Limitations

The present study also has several limitations. First of all, it uses a combination of convenience and snowball sampling, which may introduce bias toward sample homogeneity; the results depend on self-reporting, which may be biased by recall and social expectations. Secondly, since this is a cross-sectional study, it is impossible to infer causal relationships or characterize changes in individual cognition over time. Therefore, a longitudinal design can be used in future studies to understand the occupational burnout characteristics of nurses at different stages. Third, participants were recruited from a region of southwest China, which may limit the generalizability of the research results. Regional differences in health care resources, cultural norms, and support systems may shape caregivers' needs and experiences. Finally, we identified four subgroups. Although each group met the minimum participant requirement for LPA, the MLR analysis sample size was relatively small. Despite this limitation, the primary purpose of the study is to identify subgroups with distinct characteristics, thereby gaining an in-depth understanding of the type of burnout among nursing staff. In this case, the research results retain their clinical value. Future studies should use larger, more diverse samples to enhance the robustness of subgroup classification and identify additional relevant factors.

## 5 Conclusion

This study identified four potential characteristic types of professional burnout in clinical nurses: “Low Burnout - High Efficacy”, “Mild Burnout - Unfulfilled”, “Moderate Burnout - Exhausted”, and “Severe Burnout - Dysfunctional”. Factors associated with these burnout profiles included age, hospital level, years of working, as well as dimensions of the work environment such as nurse manager ability, leadership, and support for nurses and collegial nurse–physician relationships. There is a significant correlation between these factors and other factors. These findings may help us gain a deeper understanding of individual differences and focus on the specific symptoms of nurses with different types of burnout, thereby providing a basis for formulating more targeted and specific intervention strategies. For example, while paying attention to middle- and low-age nurses, providing psychological counseling and help to senior nurses, creating a good professional environment for nurses, encouraging nurses to participate in hospital affairs, reducing the burden of nurses while improving the foundation of nursing quality, and establishing a long-term support system and response mechanism to reduce the burnout of nurses.

## Data Availability

The original contributions presented in the study are included in the article/supplementary material, further inquiries can be directed to the corresponding author/s.
